# A Practical Treatise on the Diseases of the Testis, and of the Spermatic Cord and Scrotum

**Published:** 1856-09

**Authors:** 


ART. IX.—*A Practical Treatise on the Diseases of the Testis, and of the Spermatic Cord and Scrotum.* With numerous wood engravings. By T. B. CURLING, F. R. S., Surgeon to the London Hospital, etc. Second American from the Second Revised and Enlarged English Edition. Philadelphia: BLANCHARD & DEA. 1856.

Twelve years have elapsed since the first publication of this work. This second edition contains the added observations of that period, and is an amplification of the first. To make room for additional matter, the anatomical introduction was omitted in the new London Edition, but we are glad to notice that the American publishers retain it. It is by far the best account of the anatomy of the testis which we possess, and should by all means accompanying the treatise, giving it, as it does, a completeness of detail which satisfies the wants of the practitioner.

The reputation of this work has been so long and thoroughly established, that the labor of the critic may be confined to the simple announcement of its republication. Many who since the exhaustion of the previous edition have been unable to procure it, will doubtless profit by this hint. We need only add that it is the most complete, scientific, and practical account of diseases of the testis in the language.

